# Assembly and Activation of Alternative Complement Components on Endothelial Cell-Anchored Ultra-Large Von Willebrand Factor Links Complement and Hemostasis-Thrombosis

**DOI:** 10.1371/journal.pone.0059372

**Published:** 2013-03-29

**Authors:** Nancy A. Turner, Joel Moake

**Affiliations:** Department of Bioengineering, Rice University, Houston, Texas, United States of America; National Cerebral and Cardiovascular Center, Japan

## Abstract

**Background:**

Vascular endothelial cells (ECs) express and release protein components of the complement pathways, as well as secreting and anchoring ultra-large von Willebrand factor (ULVWF) multimers in long string-like structures that initiate platelet adhesion during hemostasis and thrombosis. The alternative complement pathway (AP) is an important non-antibody-requiring host defense system. Thrombotic microangiopathies can be associated with defective regulation of the AP (atypical hemolytic-uremic syndrome) or with inadequate cleavage by ADAMTS-13 of ULVWF multimeric strings secreted by/anchored to ECs (thrombotic thrombocytopenic purpura). Our goal was to determine if EC-anchored ULVWF strings caused the assembly and activation of AP components, thereby linking two essential defense mechanisms.

**Methodology/Principal Findings:**

We quantified gene expression of these complement components in cultured human umbilical vein endothelial cells (HUVECs) by real-time PCR: C3 and C5; complement factor (CF) B, CFD, CFP, CFH and CFI of the AP; and C4 of the classical and lectin (but not alternative) complement pathways. We used fluorescent microscopy, monospecific antibodies against complement components, fluorescent secondary antibodies, and the analysis of >150 images to quantify the attachment of HUVEC-released complement proteins to ULVWF strings secreted by, and anchored to, the HUVECs (under conditions of ADAMTS-13 inhibition). We found that HUVEC-released C4 did not attach to ULVWF strings, ruling out activation of the classical and lectin pathways by the strings. In contrast, C3, FB, FD, FP and C5, FH and FI attached to ULVWF strings in quantitative patterns consistent with assembly of the AP components into active complexes. This was verified when non-functional FB blocked the formation of AP C3 convertase complexes (C3bBb) on ULVWF strings.

**Conclusions/Significance:**

AP components are assembled and activated on EC-secreted/anchored ULVWF multimeric strings. Our findings provide one possible molecular mechanism for clinical linkage between different types of thrombotic and complement-mediated disorders.

## Introduction

Common clinical characteristics of the thrombotic microangiopathies, thrombotic thrombocytopenic purpura (TTP) and atypical hemolytic-uremic syndrome (aHUS), include microvascular platelet adhesion/aggregation/occlusion, thrombocytopenia, and mechanical hemolysis. [1] TTP is often associated with a deficiency of functional ADAMTS-13 (mutations or autoantibody-inhibited), the protease responsible for regulating the size of circulating VWF multimers. There is an accumulation of ULVWF strings on endothelial cell (EC) surfaces under conditions when the ULVWF strings are secreted at increased rates combined with lower amounts of functional ADAMTS-13. [2], [3] Bacterial toxins, inflammatory cytokines, phosphodiesterase inhibitors and calcium ionophore are among the agents that cause increased rates of ULVWF secretion from ECs [4]–[6]. aHUS is the result of excessive complement activation or, more commonly, defective regulation of proteins of the alternative complement pathway (AP). The primary effect of uncontrolled AP activity in aHUS is damage to renal endothelium, resulting in renal failure [7].

Although it has been established that AP regulation is dysfunctional in aHUS, it is unclear what initiates the AP activation. Limited activation of the AP can begin by direct hydrolysis of an intra-molecular bond in C3 to C3-H_2_O. Subsequent cleavage activation of C3, releasing 9 kDa fragment C3a to form C3b, and further amplification of C3b production depends on the presence of “activating surfaces” [8]. C3b (not intact C3) attaches covalently via an exposed thioester to hydroxyl-containing amino acids (threonine, serine and tyrosine) on activating surfaces [9]. C3b then binds factor B (FB) to produce C3bB [10], [11]. FB in the C3bB complex is cleaved to active Bb by factor D (FD) to produce C3bBb, the AP C3 convertase (with t_1/2_ of 1–3 min) [12] that is stabilized by factor P (properdin; FP) [13]–[15]. The Bb in C3bBb on an activating surface cleaves fluid-phase C3 to generate additional surface-bound C3b, a process that rapidly amplifies C3b generation from C3. As the ratio of C3b to Bb increases, C3bBbC3b is formed (as the AP C5 convertase), binds C5 with high affinity, and cleaves C5 to C5b [12], [16]. C5b combines with C6 and C7 to generate C5b67 complexes that insert into cell membranes. If C8 and multiple C9 molecules combine with C5b67 complexes in the cell membrane, then lytic C5b678(9)_n_ terminal complement complexes (TCCs) are formed.

Factor H (FH) and factor I (FI) are fluid-phase negative regulatory proteins of the AP [17], [18]. FH can displace Bb from C3bBb and C3bBbC3b complexes and enables FI to cleave and inactivate C3b [19]. Heterozygous mutations of the *CFH* gene or autoantibody-mediated inhibition of FH are prominent causes of aHUS [20], [21]. aHUS is also associated with heterozygous loss-of-function mutations of *CFI*, and heterozygous gain-of-function mutations in *C3* or *CFB* [22], [23].

In contrast to the AP, the classical complement pathway (CP) and lectin-activated complement pathway (LP) are initiated by C1 (complex of C1q_6_, C1r_2,_ C1s_2_) attachment to antigen-antibody aggregates or mannose/N-acetylglucosamine-binding lectin (MBL)/MBL-associated protein (MASP), respectively [24], [25]. Both the CP and LP lead to cleavage and activation of C4 and C2 to generate C4b2a complexes [8], [26]. Analogous to activated C3b, activated C4b has an exposed thioester capable of binding covalently to surfaces [10]. The C2a protease in C4b2a (the classical/lectin pathway C3 convertase) cleaves C3 into active C3b.

Human ECs of a variety of types (umbilical vein, arterial, lung microvascular, glomerular microvascular) secrete and anchor ULVWF strings in response to many stimuli [5], [27]. *In vivo*, EC-secreted/anchored ULVWF strings are exposed to all of the complement components in the circulation. After verifying and quantifying human umbilical vein endothelial cell (HUVEC) expression of complement proteins, we initially studied ULVWF strings and the attachment of complement components that were released exclusively from cultured ECs in the absence of other plasma proteins. ULVWF multimers are compressed in WPBs in a spring-like conformation that allows its rapid unfolding to the EC surface after stimulation, without additional application of shear stress or flowing conditions [28]. In our experiments, the non-flowing static conditions allowed HUVEC-released complement components to accumulate and bind to their targets [3], [29]. The extensive interactions observed between AP components and the anchored ULVWF strings suggested AP activation. To test this hypothesis, we added normal serum, heated in order to inactivate FB (a component essential for AP activation), to the stimulated HUVECs and measured the changes in C3, C5 and FB attachment to EC-anchored ULVWF strings. Our goal was to determine if HUVEC-anchored ULVWF strings function as activating surfaces capable of initiating AP component attachment and activation. The goal was achieved in our studies, and we demonstrate here for the first time a possible molecular mechanism linking complement activation and the initial events in hemostasis-thrombosis.

## Results

### HUVEC Gene Expression of Complement Components

**Figure 1:**
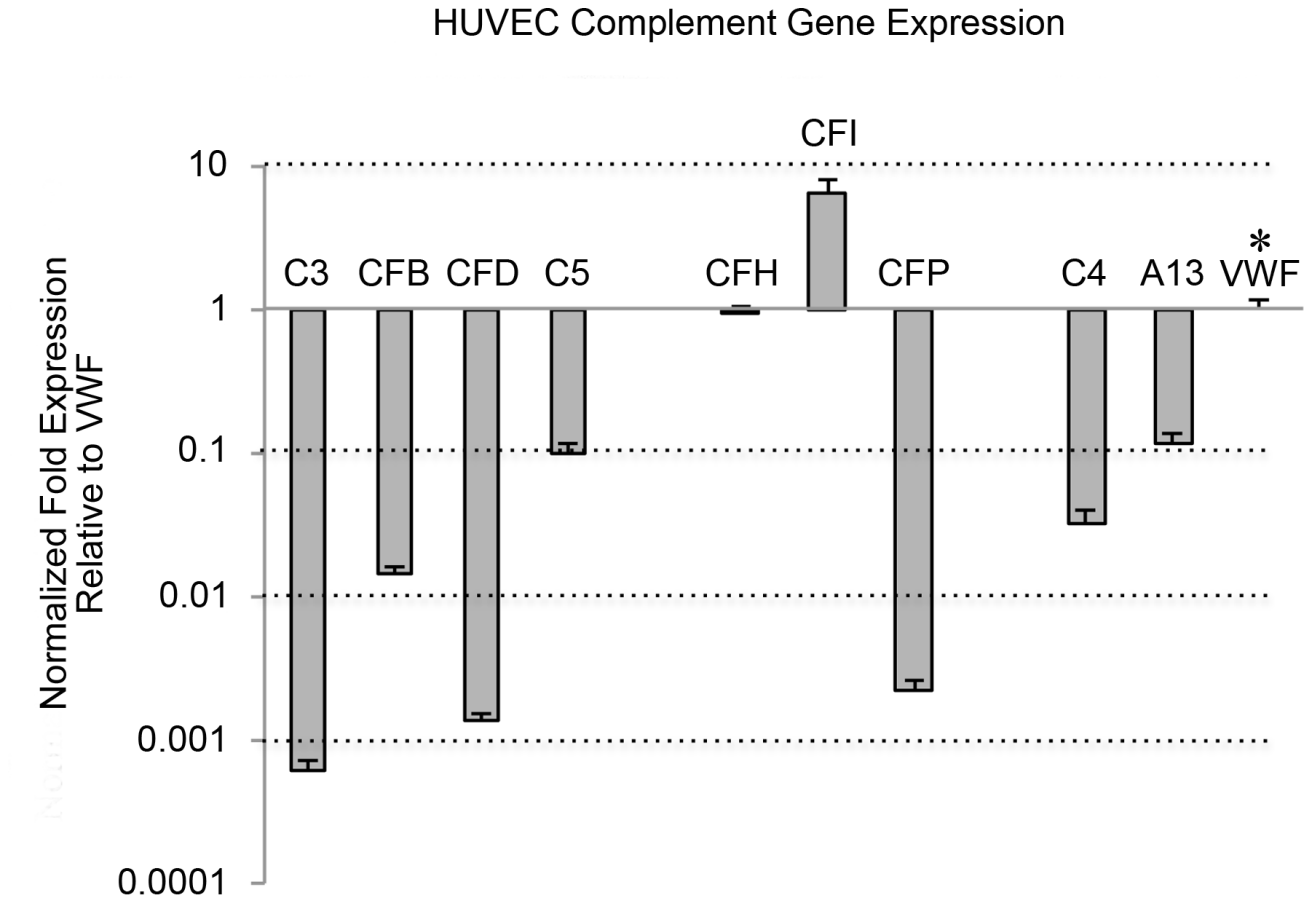
Gene expression of complement components in HUVECs. HUVECs were maintained for 24 hours in serum-free medium prior to RNA extraction. Total RNA was isolated, reverse transcribed, and the cDNA was analyzed by real-time PCR using TaqMan probes with *GAPDH* as the reference gene. The graph shows fold differences (log scale) of complement component expression in unstimulated HUVECs relative to *VWF* expression (marked by asterisk). The line at 1 is the boundary between increased and decreased expression. Data shown are means plus SD, N = 4. Values for *ADAMTS13* (A13) are shown for comparison.

Transcripts for *C3* and *C5*, the AP-specific complement components *CFB*, *CFD*, *CFP*, *CFH*, *CFI*, and the CP-specific component *C4* were identified in HUVECs and quantified relative to *VWF* expression for the first time using real time RT-PCR (**Fig. 1**). TaqMan probes that recognize only cDNA transcribed from mature mRNA were used in order to exclude genomic DNA. Synthesis of *CFD* has never previously been detected in HUVECs by any technique. FD is required to cleave C3b-bound FB to Bb [the C3 convertase (C3bBb)] during AP activation.

The alternative pathway regulatory components *CFH* and *CFI* were the only complement genes expressed in HUVECs at levels in the range of *VWF*, a major synthetic product of human ECs: *CFH* expression was similar to *VWF* and *CFI* was ∼6-fold higher. Expression levels of *C5* and *CFB* were 10-fold and 70-fold lower, respectively; and *C3*, *CFD* and *CFP* were ∼500 to 1200-fold lower than *VWF* expression levels.

Expression of the CP component *C4* was 50-fold lower than *VWF*.

Transcripts for the VWF protease, *ADAMTS13*, which is also produced and released from HUVECs, [30] were ∼10-fold lower than *VWF* transcripts. *ADAMTS13* was included in this study as an additional indicator of EC transcription.

### HUVEC-released Complement Components Bind to HUVEC Secreted and Anchored ULVWF Strings

In the presence of the EC stimulatory substance, histamine, HUVECs rapidly (within 2 min) secrete ULVWF strings from their storage vesicles [Weible-Palade bodies (WPBs)] onto cell surfaces. We have previously demonstrated the release of anchored ULVWF strings from histamine stimulated HUVECs under non-flowing, static experimental conditions. The static conditions allow the accumulation of HUVEC-released proteins that would be washed away under flowing conditions [3], [29]. Antibodies to VWF were added 2 min after the ECs were stimulated with histamine, to identify fluorescently the ULVWF strings and to prevent cleavage of the secreted/anchored ULVWF strings by HUVEC-released ADAMTS-13. Over the following 15 min complement components released from the HUVECs attached to the EC secreted/anchored ULVWF strings.

**Figure 2:**
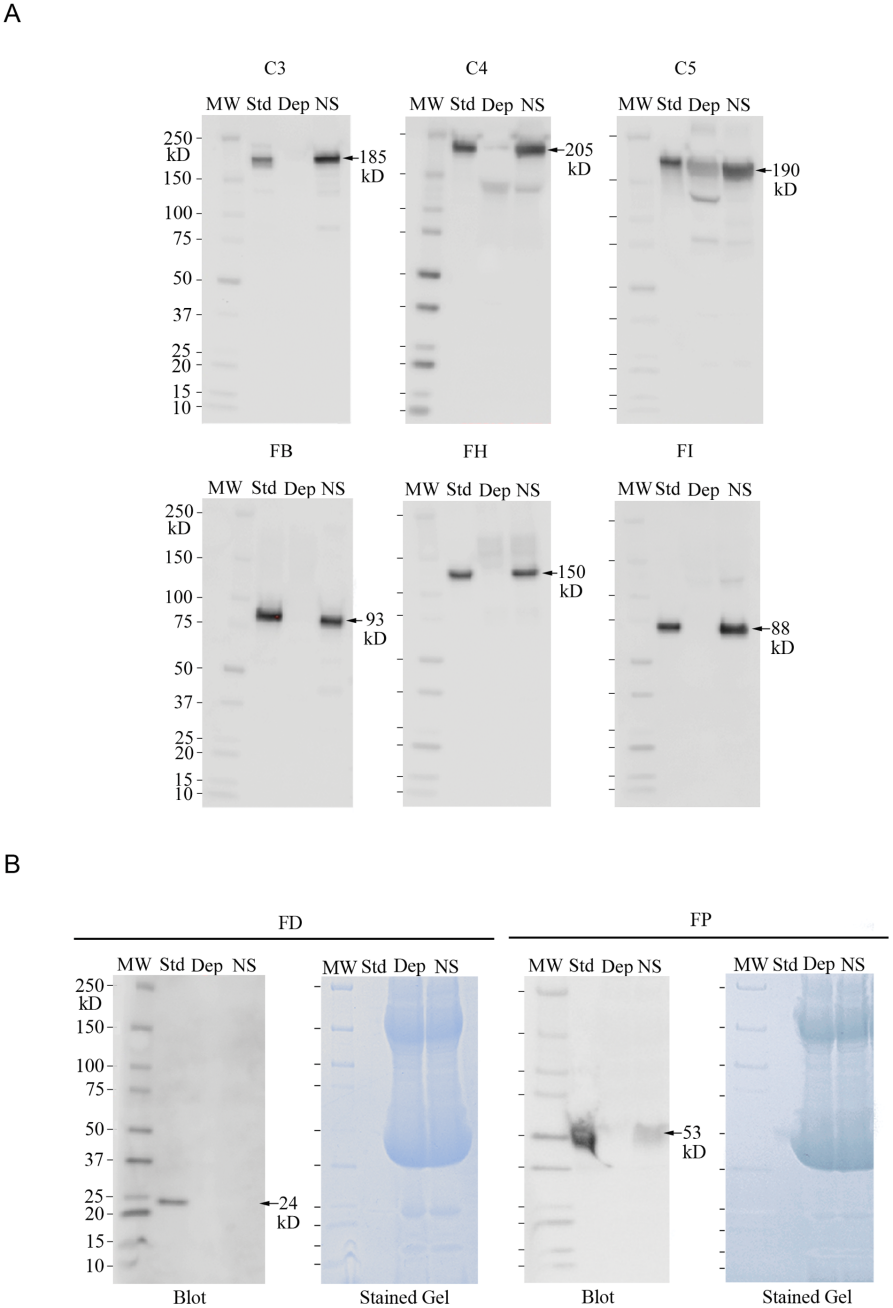
Specificity of antibodies to human complement components. (A) Denatured, non-reduced samples were separated by 4–15% SDS-PAGE and transferred blots were detected with polyclonal goat antibodies to single human complement components. Each blot contains lanes with: 50 ng of a purified complement protein (Std); normal serum (NS) containing 50 ng of the specific complement component; and an equal volume of specific complement component-depleted serum (Dep). Arrows show relative molecular mass of each protein migrating in SDS and MW indicates molecular weight markers in kDa. (B) FD and FP were analyzed by Western blots as described in (A) except: In the FD blot the Std lane contains 159 ng FD and NS lane contains 4 ng FD (FD serum conc. 1–2 ng/µl); and in the FP blot the NS lane contains 10 ng FP (FP serum conc. 4–6 ng/µl). The Coomassie stained gels show the high levels of protein (∼100 µg/lane) in the serum samples that were applied to the gels.

Fluorescent imaging was used to analyze the attachment of complement proteins to HUVEC-secreted and anchored ULVWF strings. The polyclonal antibodies made against human complement proteins used in fluorescent microscopy experiments specifically identify individual complement components, as demonstrated by Western blots (**Fig. 2**). The serum samples applied to the gels (usually 20–25 µg/lane) contained many-fold higher amounts of protein than were secreted by the HUVECs during our experiments; nevertheless, bands for other proteins other than the specific complement factors (and corresponding cleavage fragments) were not detected by the individual mono-specific polyclonal antibodies made against the different complement components. A degradation product of C4 is detected in the C4-depleted serum and two degradation products of C5 are detected in the C5-depleted serum.

In order to analyze FD and FP in serum, the quantities of protein in gel samples were increased even further to ∼100 µg/lane. This is the maximum amount of protein per gel lane that can enter completely, and be separated effectively, in our electrophoresis system. The FD in the serum samples was still undetectable because of the low FD serum concentrations (1–2 ng/µl). In the blot detected with antibody to FP, the migration of the FP standard was altered slightly by the high albumin concentration present in the FP-depleted sample in the adjacent lane. The FP in normal serum (4–6 ng/µl) was barely detectable.

The complement components in this study were not detected in HUVEC WPBs and, therefore, it is improbable that ULVWF multimers were pre-bound with complement proteins prior to their secretion. The technical details are in “Fluorescent emission ‘bleed-through’ controls” in the Material and Methods section.

**Figure 3:**
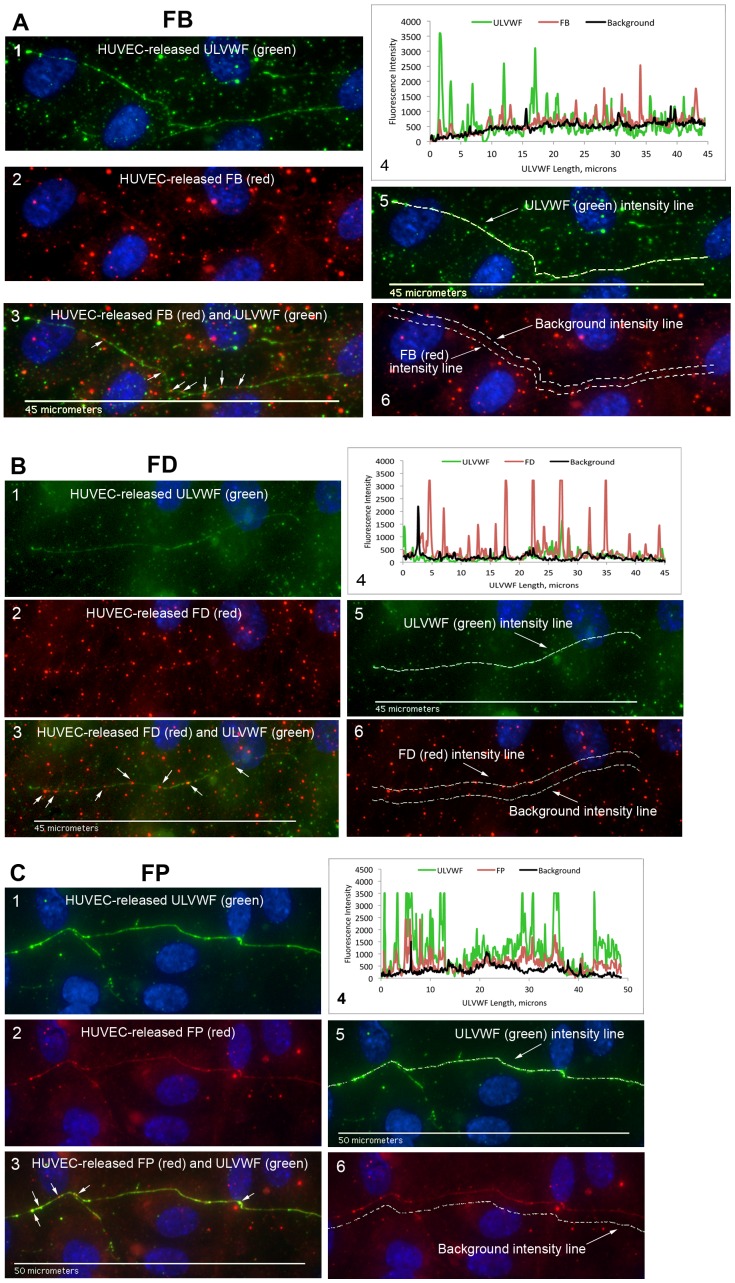
AP-specific components FB, FD and FP attach to ULVWF strings secreted by, and anchored to, stimulated HUVECs. HUVECs were stimulated with 100 µM histamine and stained with rabbit anti-VWF plus secondary fluorescent anti-rabbit IgG-488 (green). Cells were then p-formaldehyde-fixed and stained with goat IgG antibody to human FB (A), antibody to human FD (B), and antibody to human FP (C) plus secondary fluorescent anti-goat IgG-594 (red). The HUVEC nuclei were labeled with DAPI (blue). In (A) are: (1) ULVWF (488-nm, green); (2) FB (594-nm, red); and (3) ULVWF and FB combined image; (4) Graph of fluorescent intensities (y-axis) measured from identical locations in ULVWF string images (488-nm, green) and in complement component proteins images (594-nm, red) are plotted against the ULVWF string length (in microns, x-axis). The black line indicates the background intensities measured in the 594-nm images. (5) ULVWF intensities were measured along lines of ULVWF strings detected at 488-nm (shown by dotted line); (6) FB intensities were measured in 594-nm images along lines at identical locations (shown by lower dotted line) as determined in (5). Background intensities were also measured in 594-nm (red) images at parallel locations (shown by upper dotted line) away from the area of interest. Similar types of images are shown using antibody to human FD in (B) panels 1–6 and antibody to human FP in (C) panels 1–6 to identify the complement component attached to the HUVEC-secreted/anchored ULVWF strings. In (C) panel 6, only the locations of the background intensities are identified by the dotted line. The white arrows in (3) indicate FB (A), FD (B) and FP (C) attachment to the strings. Images were selected from 5 (FB and FD) and 4 (FP) independent experiments.

In the initial fluorescent imaging experiments, the complement proteins analyzed for attachment to HUVEC-secreted and anchored ULVWF strings were synthesized and released exclusively from HUVECs and accumulated under the non-flowing experimental conditions. Neither a serum nor plasma source of the components was present in the experiments. Fluorescent images and fluorescent intensity graphs of complement components attaching along the HUVEC-secreted/anchored ULVWF strings are shown in **Figs. 3** **,** **4** **,** **5** **,** **6** **,** **7**, and the quantitative attachment data are summarized in **Fig. 8**.

**Figure 4:**
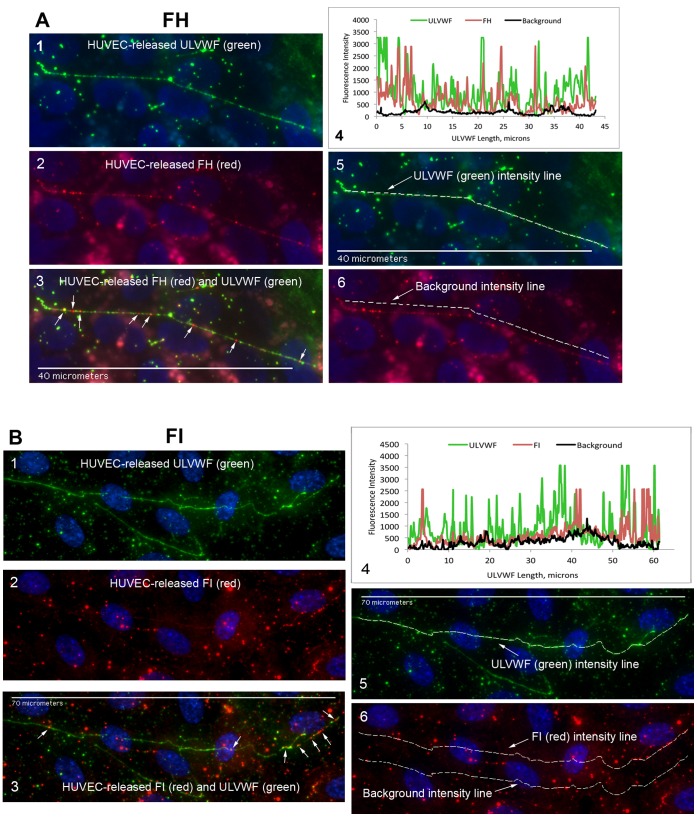
AP-specific negative regulatory components FH and FI attach to ULVWF strings secreted by, and anchored to, stimulated HUVECs. HUVECs were stimulated and stained as in the legend for Fig. 3, except that antibody to human FH was used in (A) and antibody to human FI in (B) to identify complement component attachment to the ULVWF strings. In (A) panel 6, only the locations of the background intensities are identified by the dotted line. In (B) panel 6, the upper dotted line shows the location of the FI intensity measurements and the lower dotted line identifies the locations of background intensity measurements. The white arrows in (3) indicate FH (A) and FI (B) attachment to the strings. Images were selected from 12 (FH) and 4 (FI) independent experiments.

**Figure 5:**
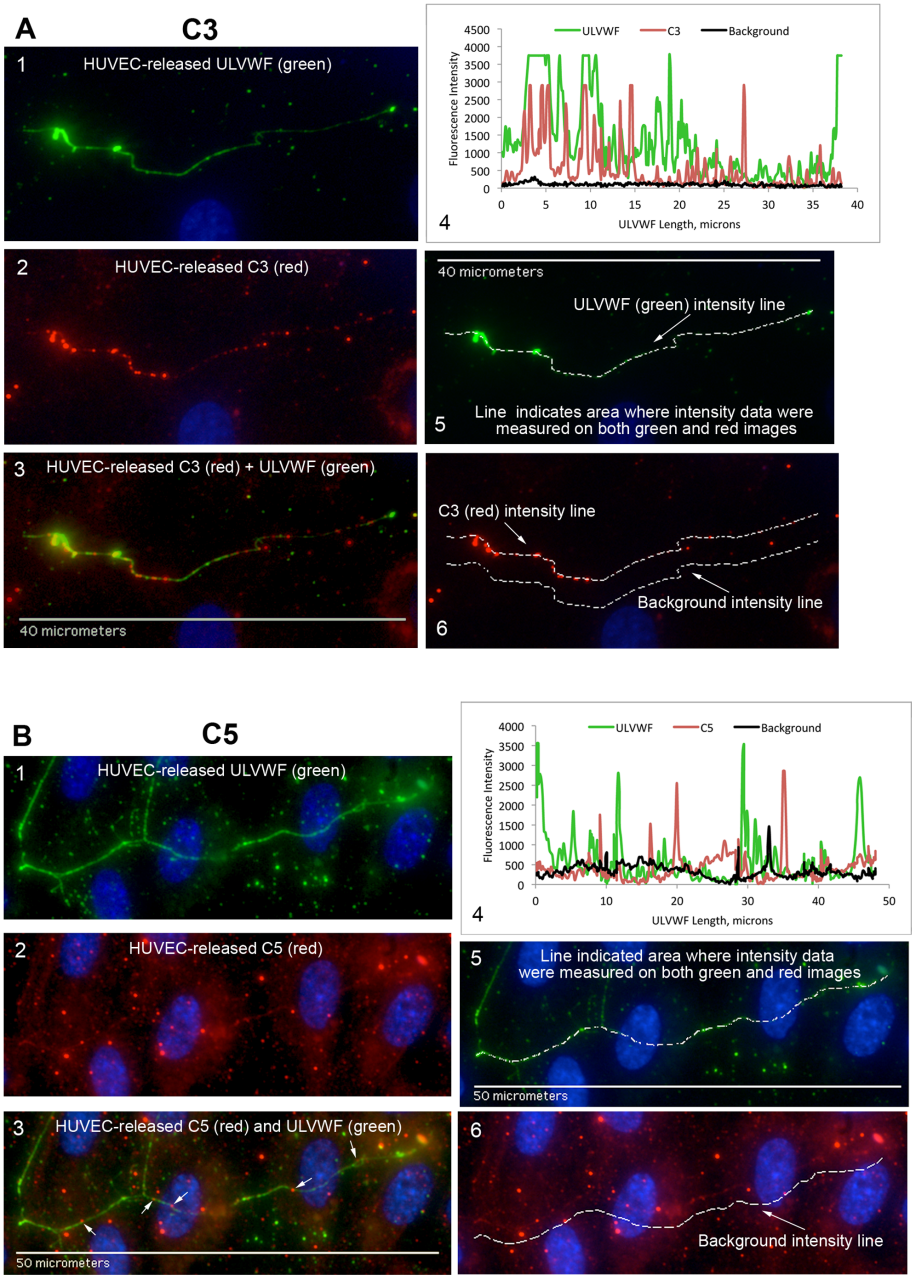
Complement components C3 and C5 attach to ULVWF strings secreted by, and anchored to, stimulated HUVECs. HUVECs were stimulated and stained as in the legend for Fig. 3, except that antibody to human C3 was used in (A) and antibody to human C5 in (B) to identify complement component attachment to the ULVWF strings. In (A) panel 6, the upper dotted line shows the location of the C3 intensity measurements and the lower dotted line identifies the locations of background intensity measurements. In (B) panel 6, only the locations of the background intensities are identified by the dotted line. The white arrows in (B) panel 3 indicate C5 attachment to the strings. Images were selected from 6 (C3) and 5 (C5) independent experiments.

**Figure 6:**
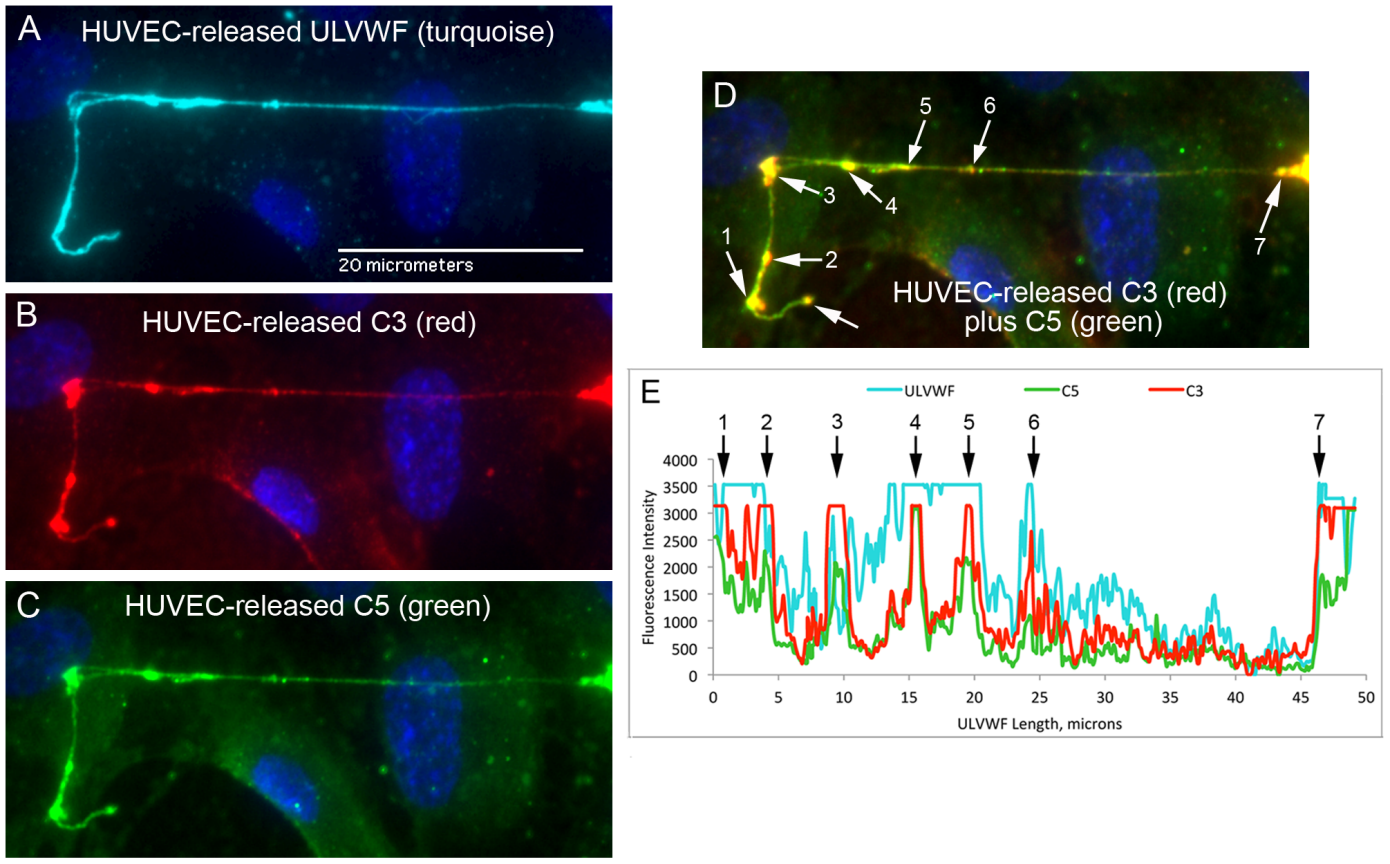
Complement components C3 and C5 attach to the same positions along HUVEC secreted/anchored ULVWF strings. HUVECs were stimulated and stained as in the legend for Fig. 3, except that the cells were simultaneously stained for C3 and C5 (in addition to VWF and DAPI). Individual fluorescent channels detected: (A) rabbit anti-VWF plus anti-rabbit IgG-488 (turquoise); (B) a combination of two mouse monoclonal antibodies to human C3 (clone 755 against C3b and clone 10A1 against C3) plus anti-mouse IgG-647 (red); and (C) goat anti-human C5 plus anti-goat IgG-594 (green). (D) Simultaneous detection of C3 (red) and C5 (green) is colored yellow in the combined image from 647- and 594-nm channels. White arrows indicate points along the ULVWF strings where high intensity levels of C3 and C5 were detected. (E) Graph of fluorescent intensities (y-axis) along the ULVWF string (488-nm, turquoise), C3 (647-nm, red) and C5 (594-nm, green) are plotted against the ULVWF string length (in microns, x-axis). The black numbered arrows correspond to the white numbered arrows in (D) and point to the C3 and C5 peak intensity locations. Images were selected from 9 experiments with simultaneous VWF, C3 and C5 staining.

**Figure 7:**
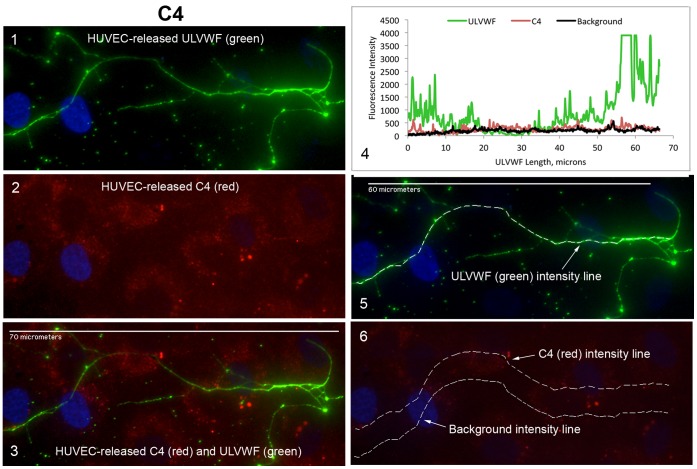
C4, a component of the classical and lectin pathways, does not attach to ULVWF strings secreted by, and anchored to, stimulated HUVECs. HUVECs were stimulated and stained as in the legend for Fig. 3, except the antibody to human C4 was used to identify complement component attachment to the ULVWF strings. In panel 6, the upper dotted line shows the location of the C4 intensity measurements and the lower dotted line identifies the locations of background intensity measurements. Images were selected from 4 experiments.

**Figure 8:**
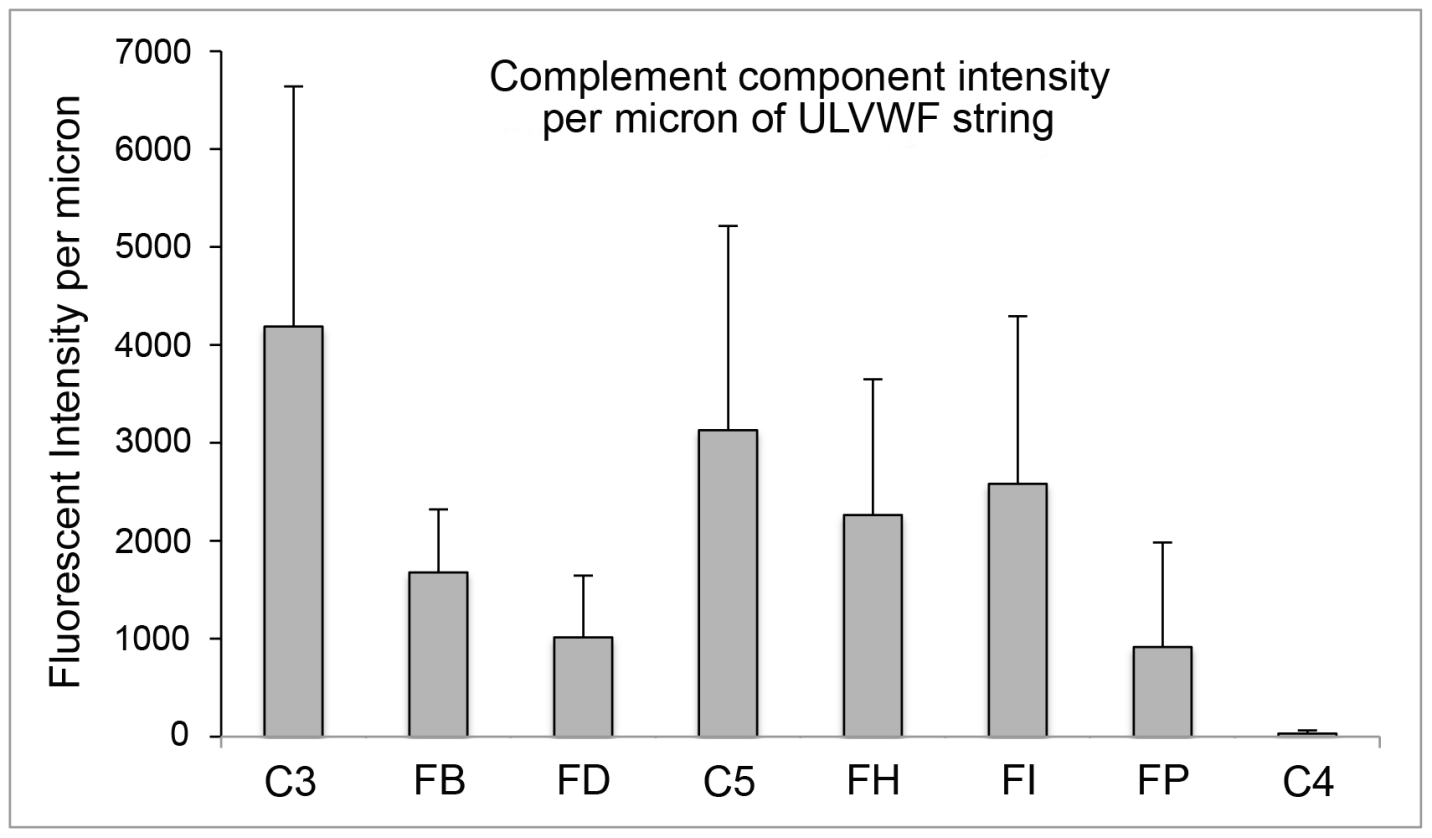
Quantification of HUVEC-released complement components attached to HUVEC-secreted/anchored ULVWF strings. Intensities of each HUVEC-released complement proteins were measured along histamine-stimulated HUVEC-secreted/anchored ULVWF strings, as described in the legend for Fig. 3. Shown are the complement component fluorescent intensities per micron of ULVWF string length after background subtraction. Values are means plus SD; N = 7–12 strings for each complement component from 4 to 12 experiments and were compiled from 130 fluorescent images. Some data were collected from images within the same experiment at a different location on the coverslip.

### AP-specific Complement Components FB, FD and FP Attach to HUVEC-secreted/anchored ULVWF Strings

Each of the AP-specific components FB, FD and FP (the positive AP regulatory protein) bound to the ULVWF strings with average fluorescent intensities per micron of ULVWF string length that were 30- to 50-fold higher than values for the classical pathway-specific component C4 (C4 data shown in Fig. 7). FB, reactive only with activated C3b not intact C3, bound most extensively to the ULVWF strings (>1600 fluorescent intensity per micron) (**Fig. 3A**). The measured fluorescent intensities for FP and FD (∼1000 units/micron) were also high, considering the low expression levels of these components in HUVECs (as shown in Fig. 1). This demonstrates a high affinity of FD and FP for HUVEC-anchored ULVWF strings (**Fig. 3B–C**).

### AP-specific Regulatory Components FH and FI Attach to HUVEC-secreted/anchored ULVWF Strings

Fluorescent intensities measured for the AP-specific negative regulatory components FH and FI along the ULVWF strings were similar to each other (**Fig. 4A–B**, ∼2400 units/micron), averaging ∼30 to 40% lower than the fluorescence measured for the most extensively bound complement components, C3 and C5, as discussed below. FH displaces FB or Bb bound to C3b, thereby preventing further AP activation. FH also acts as a cofactor for the FI proteolysis and inactivation of C3b.

### Complement Components C3 and C5 Attach to HUVEC-secreted and Anchored ULVWF Strings

HUVEC-released C3 (in the form of C3b) was the complement component that bound most extensively to the secreted/anchored ULVWF strings, with average intensities of >4000 fluorescent intensity units per micron of ULVWF string length (**Fig. 5A**). HUVECs synthesize low levels of C3, as demonstrated by *C3* mRNA levels in Figure 1; however, the extensive attachment of C3 indicates a high level of affinity of C3 for the HUVEC-anchored strings.

C5 released from HUVECs was the second most abundant complement component detected along HUVEC-anchored ULVWF strings (>3000 fluorescent intensity units per micron) (**Fig. 5B**).

The binding of C5 suggests that C5 convertases (C3bBbC3b) have formed on the ULVWF strings because C5 binds preferentially to C3b molecules within or adjacent to C3bBbC3b complexes [16]. This interpretation was confirmed by the demonstration that C3 and C5 often attached to the same positions on HUVEC-secreted/anchored ULVWF strings (**Fig. 6**). In these experiments, C3 was detected using a combination of two mouse monoclonal antibodies. One of the monoclonal antibodies was reactive only with C3b.

### Classical and Lectin Pathway Complement Component C4 Does Not Attach to HUVEC-secreted/anchored ULVWF Strings

In contrast to C3 and C5, and the AP-specific components, there was almost no binding of classical component C4 to ULVWF strings. The average C4 fluorescent intensity measured along the strings was 100-fold less per micron than the intensities measured for C3 or C5 (**Fig. 7**).

### Quantitative Summary

The quantitative data of exclusively HUVEC-released complement component binding to EC-secreted/anchored ULVWF strings is summarized in **Fig. 8**. The fluorescent intensity at 594 nm (red), used for detection of the complement proteins attached to HUVEC-anchored ULVWF strings, was not a result of fluorescent “bleed through” from the 488-nm channel (green) used for VWF detection. The experimental details that confirm this conclusion are in the Materials and Methods section.

### Complement Components do not Bind to Surfaces of Unstimulated HUVECs

**Figure 9:**
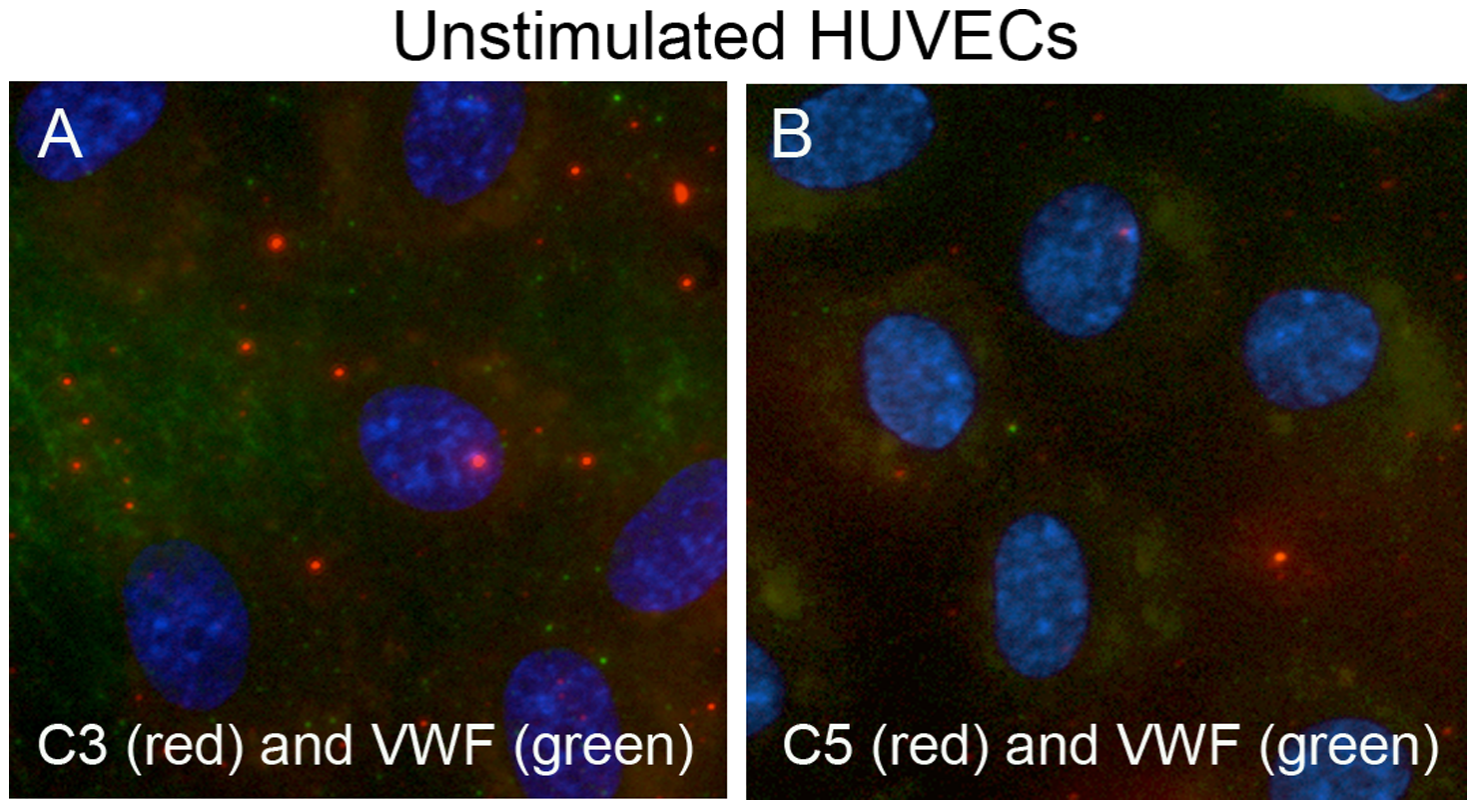
Complement components C3 and C5 do not attach to unstimulated HUVECs. HUVECs were washed once with PBS, incubated in 25% heated serum/PBS for 5 min, and washed 4X with PBS before staining with rabbit anti-VWF plus anti-rabbit IgG-488 (green), goat antibodies either to (A) C3 or (B) C5 and anti-goat IgG-594 (red). Combined images with DAPI-stained nuclei are shown at 600X and are representative of 3–4 experiments.

Neither C3 nor C5 exclusively released from the HUVECs, or added in heated serum was detected on unstimulated HUVEC surfaces devoid of ULVWF strings. The absence of C3 and C5 on HUVEC surfaces, along with the absence of anchored ULVWF strings, indicates that C3 and C5 were only bound to HUVEC-secreted/anchored ULVWF strings in our experiments (**Fig. 9**).

### Non-functional FB Reduces the Amounts of AP Components on EC-secreted/anchored ULVWF Strings: Functional Evidence for C3bBb (C3 convertase) and C3bBbC3b (C5 Convertase) Assembly

The concentrations of complement proteins in normal human serum are many-fold higher than the accumulated amounts released by the HUVECs over the 15 min time period of the previous experiments (summarized in Fig. 8). In the experiments described in this section, heated serum (diluted to 25% in PBS) was added to HUVECs during histamine stimulation. Binding intensities per micron along EC-anchored ULVWF strings was compared for C3, C5 and FB with the previous experiments using exclusively HUVEC-released complement proteins. Heating to 56°C was necessary in order to prevent heat-labile serum ADAMTS-13 from cleaving the EC-anchored ULVWF strings prior to the addition of antibody to VWF (which also blocks ADAMTS-13-mediated VWF cleavage). The functions of C3 and C5 are unaffected by 56°C heat. In contrast, heating to 56°C completely inhibits the proteolytic function of FB [31].

**Figure 10:**
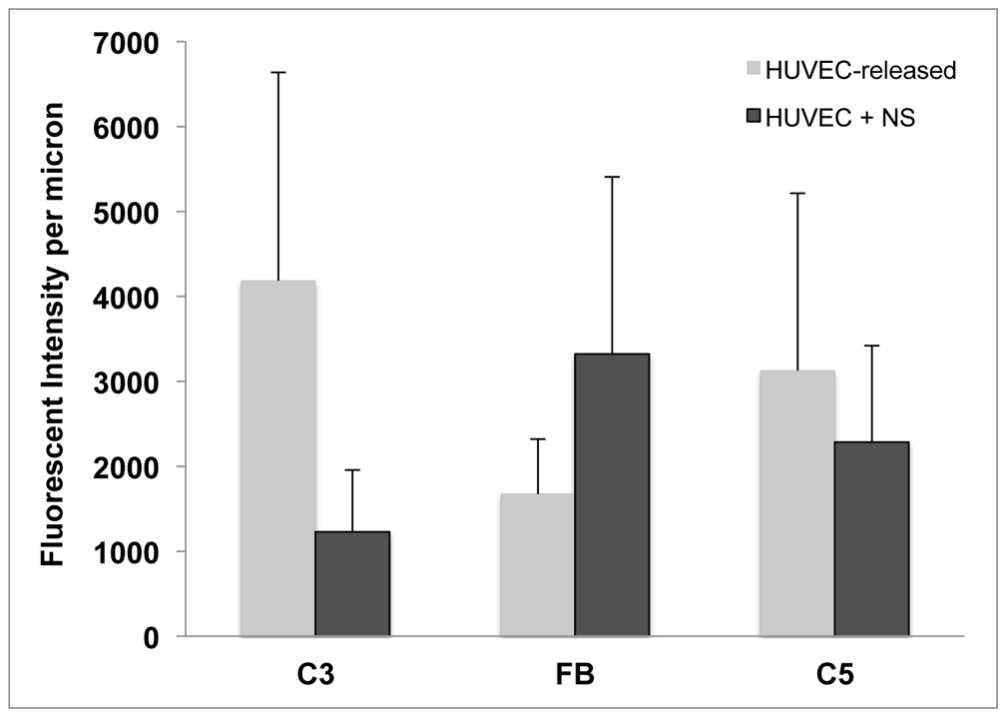
Attachment to HUVEC-secreted/anchored ULVWF strings of C3, FB and C5 released from HUVECs +/− added in heated normal serum. Intensities of C3, FB and C5 were measured along histamine-stimulated HUVEC-secreted/anchored ULVWF strings as described in the legend for Fig. 3. Light gray bars represent the binding of exclusively HUVEC-released C3, FB and C5 to ULVWF strings (shown for comparison from Fig. 8), and dark gray bars show the binding of the same components per micron of ULVWF string length in the presence of normal heated serum. Values are means plus SD; N = 8–11 strings for each complement component from 5 to 7 experiments for each of C3, FB and C5 and data were compiled from 46 fluorescent images.

The non-functional, structurally altered, heated form of FB exhibited an increased capacity for binding to HUVEC-anchored ULVWF strings. The binding intensities of non-functional FB per micron of ULVWF string in heated serum experiments were 2-fold higher than the intensities of functional FB released exclusively from HUVECs (**Fig. 10**). In contrast, less than half as much functional C3 from heated serum attached to ULVWF strings compared to the amounts of functional C3 attached exclusively from HUVECs (**Fig. 10**). These results suggest that reduced amounts of activated C3 (C3b) were generated, in the absence of functional FB, even though increased amounts of fluid-phase C3 were available in the heated serum. We conclude that heated, enzymatically-inactive serum FB bound competitively to C3b on the ULVWF strings and formed inactive C3b-FB complexes instead of active C3 convertases (C3bBb). Inactive C3b-FB complexes are incapable of the proteolytic cleavage of C3 to activated C3b that is required to attach C3b to certain surfaces (in these experiments, to HUVEC-anchored ULVWF strings).

The binding of C5 to ULVWF strings also did not increase with the addition of higher quantities of functional C5 in heated serum (**Fig. 10**). This is compatible with a reduced number of ULVWF string-bound C3b molecules restricting binding sites for C5 on C3b molecules adjacent to, or in, C3bBbC3b (C5 convertase) complexes [12], [16]. The large increase in heat-inactivated FB binding to the EC-anchored ULVWF strings may further restrict C5 binding to the ULVWF strings by sterically hindering the access of C5 to binding sites on C3b.

## Discussion

In earlier reports, HUVEC transcripts of *CFH* [32], [33] and *C5* [34] were easily identified; however, gene expression of other complement proteins was less convincing [33], [35], [36]. *CFD* expression in HUVECs had not previously been investigated. In our study, transcripts for complement components *C3*, *C4A*, *C5*, *CFB*, *CFD*, *CFH*, *CFI* and *CFP* were verified and quantified relative to *VWF* expression in unstimulated HUVECs using real-time PCR and TaqMan expression assays.

AP negative regulatory components *CFH* and *CFI* were the only complement genes expressed in HUVECs at levels comparable to *VWF*. These results indicate that EC synthesis of FH and FI are important for EC self-protection.

Excessive secretion/anchorage of endothelial cell-ULVWF strings occurs in response to endothelial cell stimulation by many agents, including histamine, shiga toxins, and inflammatory cytokines [4]–[6], [28]. Under our experimental conditions, [3] ADAMTS-13 cleavage of cell-bound ULVWF strings is diminished or delayed, allowing the AP components to attach to, and initiate C3b amplification, by the activating surfaces of the strings. In the majority of the complement/ULVWF string binding experiments in this study (Figures 3, 4, 5, 6, 7, 8), ADAMTS-13 was released from the HUVECs (along with complement components) during the 2 min histamine stimulation and the 10 min time period when the cells were incubated with the anti-VWF antibody and fluorescent secondary antibody combination. The cleavage function of the ADAMTS-13 during the 2 min stimulation was suppressed by the use of a relatively large volume of fluid surrounding the cells (1 ml per 4.8 cm^2^ of surface area) that reduced the affective concentration of released ADAMTS-13 near the surface of the HUVECs as the ULVWF strings were secreted and anchored. After the addition of the anti-VWF antibody, ADAMTS-13 was no longer capable of cleaving the (anti-VWF-coated) ULVWF strings [3], [29]. We make the analogy between restricted of ADAMTS-13 activity (allowing some ULVWF strings to remain uncleaved for our studies) and TTP or other thrombotic microangiopathies with ADAMTS-13 activity that may be inadequate for the rate of EC-secretion/anchorage of ULVWF strings (augmented by cytokines in infection or inflammation).

Stimulation of HUVECs with histamine may result in the release of other EC proteins or altered EC surface protein exposure, in addition to WPB secretion of ULVWF strings. This could account for the background binding (or cell surface binding) of some complement proteins. Our studies were restricted to the detection and measurement of complement proteins that were bound to the HUVEC-secreted/anchored ULVWF strings. Background subtraction of an equal number of data points, within the same images and in parallel locations, makes it unlikely that the measured intensities of the complement components on the ULVWF strings were the result of random fluorescent binding.

Following the rapid secretion of ULVWF from WPBs, ULVWF multimeric strings remain anchored to EC surfaces until smaller VWF multimers are released into the plasma by ADAMTS-13 cleavage of the EC-secreted/anchored ULVWF. Without an anchor point, the plasma-type small VWF multimers adopt a less accessible globular conformation. Although we did not investigate complement component interaction with plasma-type VWF multimers, it is possible that the C3b recognition sites present on EC-anchored ULVWF strings are not accessible (or less accessible) on the globular conformation of plasma-type VWF.

In our studies, brief stimulation times and addition of VWF antibodies (that block ADAMTS-13-mediated cleavage) combined to restricted the cleavage of EC-secreted/anchored ULVWF strings by HUVEC-derived ADAMTS-13. We previously demonstrated that the addition of antibodies to VWF does not prevent HUVEC-released ADAMTS-13 from attachment to EC-secreted/anchored ULVWF strings [3]. The current experiments demonstrate interactions between ULVWF strings and complement components released from stimulated HUVECs. With the exception of C4 (a component essential for CP and LP activation), each of the other AP complement components studied (C3, C5, and AP-specific proteins FB, FD, FH, FI and FP) attached to the HUVEC-anchored ULVWF strings.

Small amounts of C3 are released from many cell types (including HUVECs) and can be hydrated to an activated form (C3-H_2_O) that initiates the conversion of C3 to C3b. Cleavage of C3 releases the small C3a fragment and exposes a thioester in C3b that covalently attaches to “activating surfaces” [10]. As shown by our experiments, these include EC-anchored ULVWF strings. Binding affinities of FH for C3b decrease as a result of the structural changes that occur in C3b as it binds to an activating surface [11], [37]. The conformational changes in C3b after its attachment to cell-anchored ULVWF strings may limit the capacity of FH and FI to bind and inactivate C3b. This would favor the assembly of C3 convertase (C3bBb) by FB, FD and FP, and would allow amplification of C3 conversion to C3b and promote additional C3b attachment to the strings.

The assembly and activation of HUVEC-released AP components on EC-bound ULVWF strings would be associated with: HUVEC-released functional FB and C3b binding to each other on the ULVWF strings, followed by FB cleavage to Bb by HUVEC-released FD; the formation of string-bound C3 convertase (C3bBb) complexes; and amplification of C3b generation from C3. In the presence of heated serum, which contains high concentrations of functional C3 and non-functional FB, there was a decrease in C3b binding to HUVEC-anchored ULVWF strings compared to experiments when functional C3 and FB were released exclusively from HUVECs. We conclude that a considerable quantity of C3b binding to the strings, using HUVECs alone, was the result of string-bound C3bBb (C3 convertase) assembly and amplification of C3b generation from HUVEC-released C3. In the presence of non-functioning FB in the heated serum, a poorly functioning C3 convertase assembled on the ULVWF strings.

Detection on the EC-anchored ULVWF strings of HUVEC-released C3, FB and C5 implies that both the alternative pathway C3 convertase and the C5 convertase assemble on EC-secreted/anchored ULVWF strings. The attachment of HUVEC-released C3 (after cleavage to C3b) on ULVWF strings was ∼30% greater than the attachment of HUVEC-released C5 to the strings. As the number of C3b molecules attached to an activating surface increases to form C3bBbC3b complexes, then C5 binds with higher affinity to the accumulating C3b molecules [12], [16]. These data are compatible with the formation of some C3bBbC3b (C5 convertase) complexes capable of binding C5 on the ULVWF strings. This was demonstrated conclusively in images of C3 and C5 attached to the same points along HUVEC-secreted/anchored ULVWF strings.

The assembled C3 convertase (C3bBb) and C5 convertase (C3bBbC3b) complexes on EC-anchored ULVWF strings may generate TCCs [C5b678(9)_n_]. The C5 convertase cleaves C5 to C5b en route to the formation of C5b678 complexes, which can be inserted into cell membranes to associate with multiple C9 molecules. HUVEC membranes have CD46, thrombomodulin and DAF (decay-accelerating factor; CD55) to prevent surface C3 and C5 convertase assembly or persistence [18]. Endothelial cells also have cell surface CD59 and secrete vitronectin (S-protein) and clusterin to protect against TCC formation [18], [38]. We could not detect surface TCCs or soluble SC5b-9 complexes in our cell experiments, and we did not observe HUVEC lysis. If terminal complexes were generated during the short duration of our experiments, the amounts may have been too low to be detected by the polyclonal and monoclonal anti-SC5b-9 antibodies used in our assays. EC regulatory proteins may have protected HUVECs against lysis by any small quantities of TCCs were generated during our experiments.

Possible targets of any TCCs generated by activation of the alternative complement pathway on endothelial cell secreted/anchored ULVWF strings include microbes and injured or defective tissue (including endothelial) cells. In addition to histamine, calcium ionophore and phosphodiesterase inhibitors, ULVWF strings are secreted from endothelial cells that have been stimulated by cytokines (TNFα, IL-6, IL-8) associated with infection and inflammation [6].

We have demonstrated the interaction and probable assembly/activation of alternative complement components on endothelial cell-secreted/anchored ULVWF strings. The findings may have pathophysiological and potential therapeutic importance in thrombotic and complement-mediated inflammatory disorders, and provide one possible molecular mechanism for recent observations suggesting clinical links between different types of thrombotic microangiopathies. [39]–[42] Possible new therapy, in addition to a monoclonal antibody to C5 currently available, [43] includes blockade of the AP C3 convertase using heat-inactivated FB that is described for the first time in our report.

## Materials and Methods

### Ethics Statement

All work on human VWF, human endothelial cells including experiments in this study have been specifically approved by the Rice Institutional Review Board (IRB). Human tissues and blood samples were collected under a protocol approved by the Rice IRB. Donors provided their written informed consent to participate in the study. Protocol Name: Processing of Large von Willebrand Factor (VWF) Multimers: VWF Cleavage, Thrombosis and Platelet Aggregation, Protocol Number: 11-183E. The Rice IRB reviews protocols annually and has approved of this consent procedure and study through 5/13/2013.

### Complement Components and Antibodies

Goat polyclonal antibodies to individual human complement components, purified human complement proteins, and human sera depleted of each specific complement factor were obtained from Complement Technology (Tyler, TX). Monospecific reactivity of each complement antibody was verified by Western blotting using sets of purified complement proteins, normal sera and component-specific depleted sera. Each polyclonal complement antibody was reactive against the intact component protein and the corresponding cleavage fragments. Complement C3 was also identified using a combination of mouse anti-human C3b (clone 755) and anti-human C3 (clone 10A1) monoclonal antibodies (Pierce/Thermo Scientific) in the fluorescent microscope experiments. The average serum concentrations of complement proteins in this study are: C3 1300 µg/ml; C4 400 µg/ml; C5 75 µg/ml; FB 200 µg/ml; FD 2 µg/ml; FH 500 µg/ml; FI 35 µg/ml; and FP 5 µg/ml.

### Western Immunoblots

Denatured, non-reduced samples in sodium dodecyl sulfate (SDS) were electrophoresed into 4–15% polyacrylamide gels (BioRad), stained with Bio-Safe Coomassie G-250 and transferred to PVDF membranes. Membranes were overlaid separately with monospecific polyclonal goat antibodies to each complement component, followed by secondary rabbit anti-goat IgG-HRP plus StrepTactin-HRP conjugate and chemiluminescent reagents (WesternC, BioRad), before digital imaging (ChemiDoc XRS, BioRad). Each blot in Figure 2A contains lanes with: 50 ng of a purified complement protein (Std), normal serum (NS) containing 50 ng of the specific complement component, an equal volume of specific complement component-depleted sera and StrepTactin-labeled protein standards. Goat antibody to FD was pre-adsorbed with FD-depleted sera using a 1∶4 ratio.

### Human Umbilical Vein Endothelial Cells (HUVECs)

Primary HUVECs were isolated from umbilical veins as previously described [5]. Cells were seeded in flasks or on glass coverslips for microscopy experiments and grown in Endothelial Basal Medium (EBM, Lonza, Hopkinton, MA), supplemented with 3% penicillin-streptomycin (P/S), 0.2 mM L-glutamine and Low Serum Growth Supplement (Invitrogen). HUVECs used for RNA isolation were incubated for 24 hours in serum-free EBM plus insulin-transferrin-selenium (ITS, Invitrogen). HUVEC RNA isolated to calculate efficiencies for *CFD* and *CFP* were incubated for 24 hours ±100 µM histamine followed by 24 hours in serum-free EBM plus ITS.

### Fibroblasts

Human adult dermal fibroblasts were purchased from American Type Culture Collection (Manassas, VA) and maintained in Dulbecco’s Modified Eagle’s Medium (DMEM) plus 3% P/S, glutamine and 10% fetal bovine serum (Atlanta Biological). Prior to RNA extraction, fibroblasts were incubated for 24 hours ±100 ng/ml lipopolysaccharide (LPS, Sigma) followed by 24 hours in serum-free DMEM plus ITS ±100 ng/ml LPS.

### Relative Quantitative Gene Expression

HUVEC and fibroblast RNA was isolated using TRIzol (Invitrogen), chloroform extraction and isopropanol precipitation. RNA integrity was verified by 260/280 optical density ratios and 1%-agarose-formaldehyde electrophoresis, and was reverse transcribed using SuperScript III Supermix (Invitrogen). Samples (100 ng cDNA) were amplified in quadruplicate by real-time polymerase chain reaction (PCR) under conditions: 95°C for 3 min, 40 cycles of (10 sec at 95°C, 10 sec at 55°C, 30 sec at 72°C), and 95° for 10 sec followed by melting curves from 65° to 95°C (CFX96, BioRad). Amplified products were detected using TaqMan Gene Expression Assays (with 6-carboxyfluorescein-labeled probes that span target exon junctions) and Fast Advanced Master Mix (Life Technologies, Carlsbad, CA). Efficiencies (E) were determined by amplification of 100 ng–0.01 ng of cDNA, calculating the slope of the line after plotting the threshold cycle (C_T_) versus ng of cDNA and using equation (1) [44].
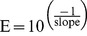
(1)

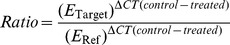
(2)


To calculate primer efficiencies, C_T_ detection of at least three 10-fold dilutions of cDNA are required for each probe. PCR amplicon for HUVEC *C3* was detected within 40 cycles with 100 ng of cDNA, but was below detection with initial amounts of 10 ng cDNA or lower. To alleviate this problem, RNA from cultured human dermal fibroblasts with/without exposure to LPS was isolated and the cDNA was used to calculate the probe efficiency for *C3*. Fibroblast expression of *C3* was 39-fold higher than in HUVECs. The addition of 100 ng/ml LPS to fibroblast cultures increased *C3* levels 19-fold further, resulting in sufficient mRNA to calculate *C3* probe efficiencies. Although HUVEC mRNA levels for *CFD* and *CFP* were comparably as low as *C3*, the transcripts for *CFD* and *CFP* increased 2- and 3-fold, respectively, in HUVECs exposed to histamine. The RNA isolated from the histamine stimulated HUVECs was used to calculate efficiencies for the *CFD* and *CFP* probes. The fold-changes in HUVEC mRNA gene expression with exposure to histamine (treated) and without histamine (control) were calculated with equation (2) using *GAPDH* as the reference gene. The standard deviation in gene expression assays (S) was determined by the equation: 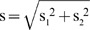 where S_1_ and S_2_ are the standard deviations of quadruplicate C_T_ measurements for the reference and target genes.

### Fluorescent Microscopy Studies

Fluorescent images were acquired using IP Lab software version 3.9.4r4 (Scanalytics, Inc., Fairfax, VA) on a Nikon Diaphot TE300 microscope equipped with a CFI Plan Fluor 60× oil N.A. 1.4 objective plus 10X projection lens (Nikon, Garden City, NY), SensiCamQE CCD camera (Cooke Corp., Romulus, MI), motorized stage and dual filter wheels (Prior) with single band excitation and emission filters for FITC/TRITC/CY5/DAPI (Chroma, Rockingham, VT). VWF and complement proteins were imaged using the following primary antibody and fluorescent secondary antibody pairs: polyclonal rabbit anti-human VWF (Ramco Laboratories, Sugarland TX) plus Alexa Fluor 488 (green) chicken anti-rabbit IgG (Invitrogen); goat polyclonal antibodies to individual human complement components plus Alexa Fluor 594 (red) donkey anti-goat IgG (Invitrogen). Complement C3 was also imaged using a combination of mouse anti-human C3b (clone 755) and anti-human C3 (clone 10A1) monoclonal antibodies (Pierce/Thermo Scientific) plus Alexa Fluor 647 goat anti-mouse F(ab’)_2_ IgG (Invitrogen). Cell nuclei (blue) were detected with 1 nM 4,6 diamidino-2-phenylindole (DAPI).

### Normal Serum

Sera from normal consenting donors collected under a protocol approved by the Rice University Institutional Review Board were pooled and stored at −80°C until use. Before cell experiments, pooled serum was heated to 56°C for 15 min (heated serum) and diluted to 25% in PBS.

### Interaction of HUVEC-secreted/anchored ULVWF Strings with Complement Components Released from HUVECs or Present in Heated Normal Serum

HUVECs seeded on 4.8 cm^2^ glass coverslips were washed with PBS and stimulated with 100 µM histamine in 1 ml of PBS for 2 min followed directly by immunostaining; or with 100 µM histamine in 25% heated serum/PBS for 5 min followed by 4 PBS washes before staining. The cytokines TNFα, IL-8 and IL-6 (+ receptor), shiga toxins-1 and -2 and histamine stimulate ECs to secrete surface-anchored ULVWF strings [5], [6]. Histamine was used in this study to stimulate the HUVECs. Followed histamine stimulation under both conditions, cells were immunostained with rabbit anti-VWF plus anti-rabbit IgG-488 for 15 min and fixed for 10 min with 1% p-formaldehyde. The fixed HUVECs were then stained separately with goat anti-human complement component antibodies plus anti-goat IgG-594 for 10 min. Cell nuclei were detected with DAPI. For unstimulated control experiments, HUVECs on coverslips were treated and stained as the heated serum-incubated cells except the histamine was omitted.

### Evaluation of HUVEC-secreted/anchored ULVWF Strings and Complement Component Interaction

HUVEC-anchored ULVWF strings detected with rabbit anti-VWF plus fluorescent anti-rabbit IgG-488 were electronically traced in 488-nm (green)-captured images at 600X magnification, and the emitted fluorescent intensity was measured and integrated along the line. The x- and y-coordinates of the traced ULVWF line were transferred to the corresponding 594-nm (red)-captured images obtained using specific polyclonal goat antibodies against single complement components plus fluorescent anti-goat IgG-594. The fluorescent intensity at 594-nm from each detected complement component was measured and integrated along the transferred line coordinates. In order to determine background 594-nm intensity, the line coordinates were trans-located ∼20 pixels (∼2.3 µm) parallel to its original position within this same image and the fluorescent intensity was measured. The quantity of each complement component (C protein) attached to the ULVWF strings was expressed as complement component intensity at 594-nm, minus the background intensity at 594-nm, divided by the ULVWF string length in microns. Image dimensions: 78 µm×58 µm, or 688 pixels×512 pixels (1 pixel = 0.113 µm).
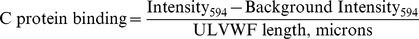



### Fluorescent Emission “Bleed-through” Controls

**Figure 11:**
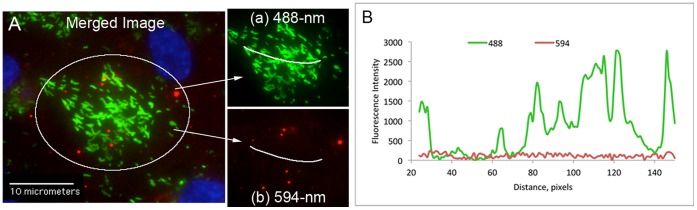
Fluorescent emission “bleed-through” controls: Weibel-Palade bodies (WPBs) contain a high concentration of VWF but are devoid of complement components. Unstimulated HUVECs were fixed with p-formaldehyde and treated with Triton-X to allow intracellular fluorescent staining. VWF in WPBs was detected with rabbit anti-VWF plus anti-rabbit IgG-488; and FB was detected with goat anti-FB plus anti-goat IgG-594. (A) The merged image was combined from 488-nm (green) and 594-nm (red) channels at 600X magnification. Single channel emissions of the circled area are shown in the inset images: (a) 488-nm and (b) 594-nm. (B) Graph of fluorescent intensities measured at points along the white lines in inset images (a) and (b) shows that extremely low intensities at 594-nm were measured at the same locations as high intensities were measured in the 488-nm channel, *i.e.*, there was little or no green-to-red “bleed-through”.

We did not detect any of the complement components in HUVEC Weibel-Palade bodies. For C3, this is in agreement with Misumi, *et al*., who previously showed that precursor C3 protein, after furin cleavage, is not sorted to a storage vesicle [45]. Unstimulated HUVECs were fixed and treated with Triton-X to allow intracellular staining, and then immunostained with anti-VWF antibody plus 488-secondary antibody. VWF staining was followed by addition of each complement antibody plus 594-secondary antibody in separate experiments. Because WPBs contain a high concentration of VWF and are devoid of complement components, these organelles were used to demonstrate the separation of fluorescent signals obtained at 488 and 594 nm in our microscope system. The fluorescent intensity at 594 nm (red), used for detection of the complement proteins attached to HUVEC-anchored ULVWF strings, was not a result of fluorescent “bleed through” from the 488-nm channel (green) used for VWF detection. This was demonstrated by the following experiments. Non-stimulated HUVECs were treated with 0.02% Triton-X to allow internal WPB staining, followed by: (1) staining with rabbit antibodies to VWF plus secondary anti-rabbit IgG-488; and (2) goat antibodies to AP components plus secondary anti-goat IgG-594. Intensities were measured across WPBs located by high levels (up to 2500 fluorescence intensity units) of VWF-positive fluorescence in 488-nm images (green), and in identical locations in 594-nm (red) images. The levels measured in the 594-nm channel were <100 fluorescence intensity units per micron, confirming that there was little or no fluorescent “bleed through” during image acquisition for the experiments with ULVWF strings (green) and the different complement components (red) (**Fig. 11**).
